# Glucocorticoid hypersensitivity as a rare but potentially fatal side effect of paediatric asthma treatment: a case report

**DOI:** 10.1186/1752-1947-2-186

**Published:** 2008-06-02

**Authors:** Sylvia Lehmann, Hagen Ott

**Affiliations:** 1 Department of Paediatrics, University Hospital Aachen, Pauwelsstrasse, D-52074 Aachen, Germany; 2 Department of Dermatology and Allergology, University Hospital Aachen, Pauwelsstrasse, D-52074 Aachen, Germany

## Abstract

**Introduction:**

Immediate-type hypersensitivity to glucocorticosteroids is rare but well known among allergists. Surprisingly, very few reports of glucocorticosteroid hypersensitivity in children exist although glucocorticosteroid treatment is particularly common in this age group.

**Case presentation:**

We report the case of a 2-year-old boy who developed generalized urticaria, facial angio-oedema, nausea and severe dyspnoea after intravenous application of prednisolone-21-hydrogen succinate. Skin prick testing with prednisolone-21-hydrogen succinate elicited a positive result; no reactions were observed to prednisone, betamethasone or dexamethasone. While fluorescence enzyme immunoassay analysis revealed no specific IgE antibodies against prednisolone-21-hydrogen succinate, CD63-based basophil activation testing with the culprit drug prednisolone-21-hydrogen succinate was positive. In contrast, additional incubation of basophils with prednisone, betamethasone and dexamethasone did not elicit any significant response. Hence, we performed an oral provocation test with betamethasone and a titrated intravenous dexamethasone challenge. As both drugs were tolerated without any complications they were recommended for future treatment.

**Conclusion:**

In a child with confirmed immediate-type hypersensitivity to glucocorticosteroids, it is still not possible to predict which glucocorticosteroid might be tolerated by solely relying on clinical history or results of skin and *in vitro *testing. Therefore, incremental glucocorticosteroid challenges under standardized clinical conditions remain necessary in order to facilitate a patient-tailored emergency treatment and to avoid severe reactions to glucocorticosteroids in these patients.

## Introduction

Immediate-type hypersensitivity to glucocorticosteroids (GCs) is rare but has been well known among allergists since the introduction of these agents in the early 1950s. To date, approximately 100 clinical cases of GC hypersensitivity have been published with a majority of patients being adults suffering from anaphylactic symptoms within several minutes of oral or intravenous GC application [1]. Surprisingly, very few reports on GC hypersensitivity in children exist although treatment with GCs is particularly common in this age group [2,3].

## Case presentation

We report the case of a 2-year-old boy who had been on a well-tolerated long-term therapy of 100 μg inhaled fluticasone-dipropionate daily for frequently recurring episodes of asthmatic exacerbations. He had intermittently received prednisone suppositories (Rectodelt™) for acute bronchopulmonary obstruction with no occurrence of adverse events and had never been given any other glucocorticoid preparations. However, a few weeks before presentation to our department, the patient was admitted to another hospital because of severe bronchospasm. As neither inhalant bronchodilators nor 100 mg of rectally applied prednisone resulted in any significant symptom relief, 50 mg of prednisolone-21-hydrogen succinate (PSH) (Solu-Decortin™) was administered intravenously. Within a few minutes the boy developed generalized urticaria, facial angio-oedema, nausea and severe dyspnoea requiring nasal oxygen supplementation. PSH medication was interrupted and symptoms spontaneously resolved within 30 minutes.

Upon presentation at our department of Paediatric Allergology following a 3-week wash-out period, the boy was in good health and we performed skin prick testing with a panel of commercially available glucocorticoids according to European Academy of Allergology and Clinical Immunology guidelines [4]. Briefly, each drug was prepared according to the manufacturer's recommendations and the respective test reagent was diluted with sterile physiologic saline solution. Epidermal testing was performed with titrated dilutions (1:100 to 1:10) and, if negative, with the pure reagent. As the drug preparation under investigation only contained the active ingredient (PSH) no further skin prick tests with additives or other excipients were initiated.

Testing with PSH at a dilution of 1:10 elicited a positive result (wheal diameter 6 mm), whereas no reactions were observed to prednisone (Rectodelt™), betamethasone (Celestamine N liquidum™) or dexamethasone (Fortecortin™). While fluorescence enzyme immunoassay (FEIA) analysis revealed no specific IgE antibodies against PSH, CD63-based basophil activation testing with PSH induced a significant increase in CD63-positive basophils as compared with controls. In contrast, additional incubation of basophils with prednisone, betamethasone and dexamethasone did not elicit any significant response.

In order to rule out any further clinically relevant hypersensitivity reactions to other GCs and to provide the patient with a safe GC-based emergency medication, we performed an oral provocation test with betamethasone and a titrated intravenous dexamethasone challenge. As both drugs were tolerated without any complications, they were recommended for further treatment.

## Discussion

The precise pathomechanism of immediate-type hypersensitivity to GCs remains unknown although the majority of authors assume that it is possibly IgE-mediated [3]. While the relevant hapten has not been identified conclusively, the cortisol metabolite glyoxal and the succinate esters of hydrocortisone and methylprednisolone have been pathogenetically implicated. These molecules are thought to possess a particularly high allergenic potential because of their increased water solubility and binding affinity to serum proteins. The clinical relevance of these findings is corroborated by the fact that hydrocortisone and methylprednisolone succinate esters have been reported to be the most common causative agents in patients with GC hypersensitivity [5]. While our patient revealed no cross-reactions with other GCs these have been described previously in other affected individuals, although no specific pattern of cross-reactivity has yet been established [6]. Furthermore, sensitization to additives and other excipients, particularly carboxymethylcellulose, has been observed in patients with GC hypersensitivity [7]. However, in the case of our patient, the applied culprit drug preparation did not contain any additives or excipients according to the information supplied by the manufacturer and the German national drug manual. In addition, the other corticosteroid reagents containing a set of additives were well tolerated during oral provocation testing.

The clinical diagnosis of GC hypersensitivity is still primarily based on a thorough medical history and skin prick testing, while conventional *in vitro *diagnostic tools are thought to have a rather low diagnostic yield. Specific IgE antibodies to corticosteroids and their respective metabolites have only rarely been described in patients with anaphylactic reactions and, as in our patient, a negative FEIA result does not exclude glucocorticoid hypersensitivity. Still, several investigators have recently suggested that the basophil activation test (BAT) might be a helpful tool in drug allergy research complementing routine tests, particularly if no proof of allergen-specific IgE is obtained by routine methods or if no commercially available *in vitro *assays exist [8]. To the best of our knowledge, this is the first report of a positive BAT in a patient with GC hypersensitivity, implying that this *in vitro *diagnostic tool may enhance detection of GC-specific immediate-type sensitization in the future.

## Conclusion

In a child with confirmed immediate-type hypersensitivity to GC, it is still not possible to predict which GC might be tolerated by relying solely on clinical history or results of skin and *in vitro *testing. Hence, incremental GC challenges under standardized clinical conditions remain necessary in order to facilitate a patient-tailored emergency treatment and to avoid severe reactions to GCs in these patients [9].

## Abbreviations

BAT: basophil activation test; FEIA: fluorescence enzyme immunoassay; GC: glucocorticosteroid; PSH: prednisolone-21-hydrogen succinate.

## Competing interests

The authors declare that they have no competing interests.

## Consent

Written informed consent was obtained from the patient's next-of-kin for publication of this case report. A copy of the written consent is available for review by the Editor-in-Chief of this journal.

## Authors' contributions

SL was involved in reviewing the literature and proof reading of the manuscript, HO was involved in collecting patient details, reviewing the literature and drafting the manuscript as the main author. Both authors have read and approved the final manuscript.

## References

[B1] Kamm GL Hagmeyer KO Allergic-type reactions to corticosteroids Ann Pharmacother 1999 33 451 460 10.1345/aph.18276 10332537

[B2] Peng YS Shyur SD Lin HY Wang CY Steroid allergy: report of two cases J Microbiol Immunol Infect 2001 34 150 154 11456363

[B3] Venturini M Lobera T del Pozo MD Gonzalez I Blasco A Immediate hypersensitivity to corticosteroids J Investig Allergol Clin Immunol 2006 16 51 56 16599249

[B4] EAACI guidelines Allergen standardization and skin tests Allergy 1993 48 48 82 10.1111/j.1398-9995.1993.tb04698.x 8342740

[B5] Burgdorff T Venelmalm L Vogt T Landthaler M Stolz W IgE-mediated anaphylactic reaction induced by succinate ester of methylprednisolone Ann Allergy Asthma Immunol 2002 89 425 428 12392389 10.1016/S1081-1206(10)62046-7

[B6] Ventura MT Calogiuri GF Matino MG Dagnello M Buquicchio R Foti C Corato R Alternative glucocorticoids for use in cases of adverse reaction to systemic glucocorticoids: a study on 10 patients Br J Dermatol 2003 148 139 141 10.1046/j.1365-2133.2003.05061.x 12534608

[B7] Caduff C Reinhart WH Hartmann K Kuhn Immediate hypersensitivity reactions to parenteral glucocorticoids? Analysis of 14 cases Schweiz Med Wochenschr 2000 130 977 983 10994063

[B8] Sanz M Gamboa PM Antepara I Flow cytometric basophil activation by detection of CD63 expression in patients with immediate-type hypersensitivity reactions to betalactam antibiotics Clin Exp Allergy 2002 32 277 286 10.1046/j.1365-2222.2002.01305.x 11929494

[B9] Rodrigues-Alves R Spinola-Santos A Pedro E Branco-Ferreira M Pereira-Barbosa M Immediate type hypersensitivity to corticosteroids: finding an alternative J Investig Allergol Clin Immunol 2007 17 284 285 17694710

